# TLR7 Agonist GS–9620 Combined with Nicotinamide Generate Viral Reactivation in Seronegative SHIV_SF162P3_-Infected Rhesus Monkeys

**DOI:** 10.3390/biomedicines11061707

**Published:** 2023-06-14

**Authors:** Zhe Cong, Yuting Sun, Cui Dang, Chenbo Yang, Jingjing Zhang, Jiahan Lu, Ting Chen, Qiang Wei, Wei Wang, Jing Xue

**Affiliations:** 1 MOH Key Laboratory of Human Disease Comparative Medicine, Beijing Key Laboratory for Animal Models of Emerging and Remerging Infectious Diseases, Comparative Medicine Center, Institute of Laboratory Animal Science, Chinese Academy of Medical Sciences and Peking Union Medical College, Beijing 100021, China; 2 Center for AIDS Research, Chinese Academy of Medical Sciences and Peking Union Medical College, Beijing 100730, China; 3 Zhejiang Cancer Hospital, Hangzhou Institute of Medicine (HIM), Chinese Academy of Sciences, Hangzhou 310022, China

**Keywords:** GS–9620, nicotinamide, latency-reversing agent, SHIV_SF162P3_, rhesus macaques, CD8^+^ T cell response

## Abstract

Antiretroviral therapy is capable of inhibiting HIV replication, but it fails to completely achieve a cure due to HIV persistence. The commonly used HIV cure approach is the “shock and kill” strategy, which employs latency-reversing agents to trigger viral reactivation and boost cellular immunity. Finding the appropriate drug combination for the “shock and kill” strategy would greatly facilitate clinical trials. The toll-like receptor (TLR) 7 agonist GS–9620 and nicotinamide (NAM) are reported as potential latency-reversing agents. Herein, we found the absence of viral reactivation when SHIV_SF162P3_-aviremic rhesus macaques were treated with GS–9620 monotherapy. However, our findings demonstrate that viral blips emerged in half of the macaques treated with the combination therapy of GS–9620 and NAM. Notably, an increase in the reactivation of the replication-competent latent virus was measured in monkeys treated with the combination therapy. These findings suggest that the GS–9620 and NAM combination could be used as a multipronged HIV latency stimulation approach, with potential for optimizing antiviral therapy design.

## 1. Introduction

The vast majority of HIV-infected patients rely on antiretroviral therapy (ART) to effectively suppresses HIV replication. However, treatment is lifelong, and is not able to achieve an HIV cure [1,2]. HIV infection remains incurable, since it establishes latent viral reservoirs, which in turn can stochastically begin reproducing viral particles once antiretroviral therapy (ART) is interrupted throughout the patient’s lifetime. The persistence of HIV reservoirs constitutes a major barrier to curing HIV infection with current ART [3,4,5]. The “shock and kill” strategy aim to reverse viral latency by using latency-reversing agents (LRAs) and following a combination of immune-mediated clearance and HIV-induced cytolysis to eliminate viral reservoirs, which is considered one of the predominant strategies for achieving an HIV cure [1,6,7].

Efforts to seek potent LRAs capable of effectively reactivating viral reservoirs are urgently needed to clear latently infected cells through host immunity and infected cell death, with the hope of achieving a non-rebound of viral load after antiretroviral therapy (ART) withdrawal. To date, a large amount of research has shown that toll-like receptor (TLR) agonists favor the “shock and kill” strategy, which stimulates antiviral immunity and induces HIV latency reversal in infected cells [8,9]. Among the TLR agonists, the highly selective TLR7 agonist GS–9620 is a promising LRA candidate due to its potential dual effects, which can not only activate HIV production from viral reservoirs, but also enhance the immune response that targets latently infected cells to control HIV infection [10,11,12,13]. Previous work in SIV- and SHIV-infected macaques has shown that the administration of therapeutic vaccination or a broadly neutralizing antibody, together with GS–9620, enhanced virologic control and delayed viral rebound [14,15,16,17].

Nicotinamide (NAM), a form of vitamin B3, is a promising LRA which can reactivate HIV production from latent reservoirs [18]. At the 23rd International AIDS Conference, Ricardo Diaz presented compelling data on the São Paulo patient—the third person who is known to have been cured of HIV. Following the cessation of antiretroviral (ARV) therapy and the administration of NAM, this patient did not experience any rebound of the virus. However, this patient did experience viral blips during treatment, indicating that NAM is an effective LRA for releasing HIV reservoirs, thereby contributing to an HIV cure. The case of the São Paulo patient is highly significant due to the favorable biological safety and viral reactivation observed in this individual. Consequently, NAM is viewed as a highly promising LRA for clinical use in the treatment of HIV.

Several novel LRAs under investigation certainly shed new light on HIV cure therapies [19,20]. In this study, seronegative SHIV_SF162P3_-infected rhesus monkeys were used to evaluate the efficacy of HIV reactivation with either GS–9620 alone or a combination of GS–9620 and NAM therapy.

## 2. Materials and Methods

### 2.1. Study Design

**Figure 1:**
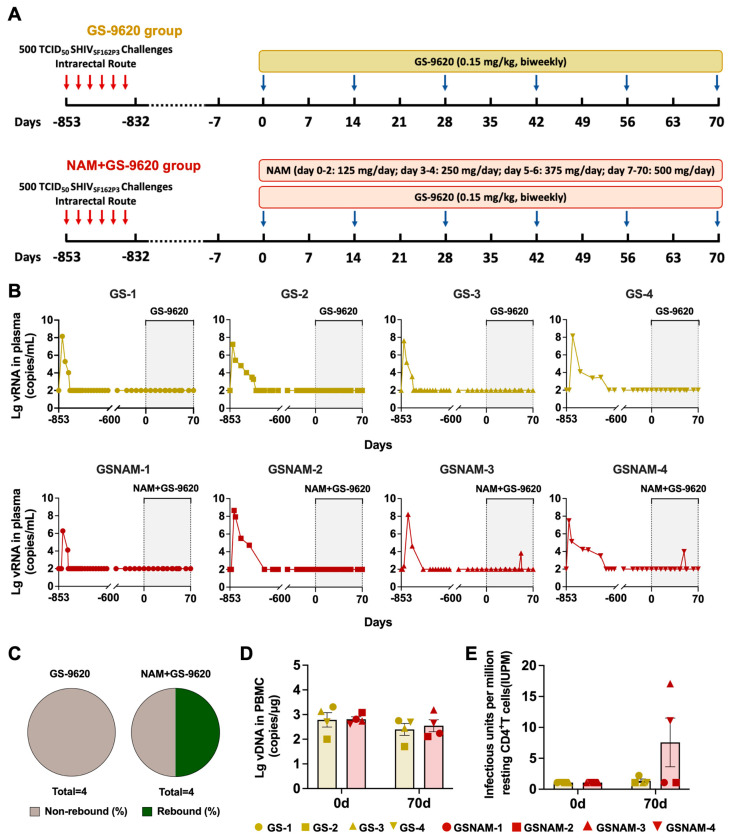
Combination of GS–9620 and NAM boosts viral reactivation in seronegative SHIV_SF162P3_-infected rhesus macaques. (**A**) Study design. A total of eight rhesus macaques were infected with 500TCID_50_ SHIV_SF162P3_ through rectal route and maintained plasma viral loads below the limit of detection for more than 20 months spontaneously. All rhesus macaques were randomly divided into two groups; four rhesus macaques in one group (GS–9620 group) were only oral administered GS–9620 (0.15 mg/kg) biweekly, while the remaining four rhesus macaques in the other group (NAM+GS–9620 group) were oral administered GS–9620 biweekly and NAM everyday (day 0–2, 125 mg/day; day 3–4, 250 mg/day; day 5–6, 375 mg/day; day 7–70, 500 mg/day) for 70 days. (**B**) Plasma viral loads of SHIV_SF162P3_-infected monkeys treated with GS–9620 or combined with NAM. The shaded area designates the period for drug treatment. (**C**) The frequency of SHIV_SF162P3_-infected rhesus macaques with virologic rebound post drug treatment among GS–9620 group and NAM+GS–9620 group. (**D**) Viral DNA in the PBMC samples of monkeys from two groups at day 0 and 70 after drug administration. (**E**) The frequency of latently infected PBMCs from all infected rhesus macaques, expressed as IUPM CD4^+^ T cells, was measured by TZA assay with a limit of detection of 1.08 IUPM.

SHIV_SF162P3_-infected Chinese rhesus macaques (*Macaca mulatta*) exhibited spontaneous virologic control without ART and were enrolled as preventive control models from previous experiments (Approval No. XJ19007). Table 1 shows the basic information for eight 6- to 9-year-old animals. All animals were negative for major histocompatibility complex class I (MHC-I) Mamu-A*01, Mamu-A*02, Mamu-B*08, and Mamu-B*17 alleles associated with the control of SIV/SHIV replication and negative for simian immunodeficiency virus (SIV), simian type D retrovirus, simian T-lymphotropic virus, and herpes B virus infections. All macaques were challenged with 500 TCID_50_ SHIV_SF162P3_ twice weekly for three weeks by intrarectal route. The animals were housed and handled in accordance with the standards of the Association for the Assessment and Accreditation of Laboratory Animal Care, and the study was reviewed and approved by the Institutional Animal Care and Use Committee (IACUC) of the Institute of Laboratory Animal Science, Chinese Academy of Medical Sciences (Approval No. XJ21006). In the first treatment stage, drug treatments began in the eight SHIV_SF162P3_-infected macaques that maintained negative plasma viral load for more than 20 months. Eight macaques were randomly divided into two groups; one group received GS–9620 (0.15 mg/kg) biweekly by oral administration for 70 days, while the other group received GS–9620 (0.15 mg/kg) biweekly combined with nicotinamide (NAM, day 0–2, 125 mg/day; day 3–4, 250 mg/kg; day 5–6, 375 mg/kg; day 7–70, 500 mg/kg) everyday by oral administration for 70 days (Figure 1A). The macaques continued to be treated for 21 days in the second treatment stage following the first treatment stage. The frequency of GS–9620 oral administration for all macaques in the two groups increased from once every two weeks to twice a week. Moreover, as for macaques receiving dual drug treatment, they were still oral administrated with NAM (500 mg/kg) every day.

### 2.2. Sample Collection and Processing

Peripheral blood from all monkeys was collected weekly for 112 days in the first treatment study, and then twice a week for 21 days and weekly for next 7 days in the second treatment study. Peripheral blood mononuclear cells (PBMCs) were isolated using standard Ficoll-density gradient centrifugation. PBMCs were cryopreserved in liquid nitrogen for long-term storage.

### 2.3. TaqMan PCR

Plasma viral RNA was extracted by using the QIAmp Viral RNA Mini Kit (QIAGEN, Valencia, CA, USA). Viral DNA was extracted from PBMCs using the DNeasy Blood & Tissue Kit DNA Mini Kit (Qiagen, Valencia, CA, USA). Plasma viral RNA and viral DNA of PBMCs were quantitated by TaqMan PCR on an ABI 7500 Real-time PCR system (Applied Biosystems, Foster City, CA, USA) [21,22]. The sequences of primers and probe were targeted against SIV-gag91 as follows: 5′- GCAGAGGAGGAAATTACCCAGTAC- 3′ (forward), 5′-CAATTTTACCCAGGCATTTAATGTT-3′ (reverse), and 5′-(FAM)-ACCTGCCATTAAGCCCGA-(MGB)-3′ (probe). The thermal parameters were as follows: 48 °C for 30 min, 95 °C for 10 min, 95 °C for 15 sec (40 cycles), and 60 °C for 1 min.

### 2.4. TZA Assay

Replication-competent viruses in resting CD4^+^ T cells were determined by TZA assay. Briefly, resting CD4^+^ T cells (CD25^−^CD69^−^HLA-DR^−^ CD4^+^ T cells) in PBMCs from SHIV_SF162P3_−infected macaques were purified by magnetic bead depletion (using CD4^+^ T cell Isolation Kit and CD69 Antibody, CD25 MicroBeads, HLA−DR MicroBeads; Miltenyi Biotec, Bergisch, Germany). Isolated resting CD4^+^ T cells were stimulated with T Cell Activation/Expansion Kit (Miltenyi Biotec) in X-VIVO 15 culture medium supplemented with 10% fetal bovine serum (FBS, Gibco, Carlsbad, CA, USA) as well as 100 U/mL IL-2 (R&D Systems, Tustin, CA, USA) and EFV (300 nM) for 6 days. The stimulated resting CD4^+^ T cells were serially diluted 4-fold (60,000—234 cells per well) and seeded in a 96-well plate containing 30,000 TZM-bl cells per well in RPMI 1640 supplemented 10% FBS. The two types of cells were co-cultured for 66 h. Wells in 96-well plate only containing TZM-bl cells were set as the negative group. Then, cells were washed and incubated with Promega β-glo for 45 min–1 h in the dark, and the chemiluminescence activity was measured by a semi-automatic chemiluminescence immunoanalyzer (Zhongsheng Baike Company, Beijing, China). Control resting CD4^+^ T cells from uninfected macaques were treated in parallel. The chemiluminescence activity from serially diluted wells was calculated after subtracting the negative control background. Then the frequency of latently infected cells among the resting CD4^+^ T cells from SHIV_SF162P3_-infected macaques were calculated using an online infectious unit per million cells (IUPM) Stats v1.0 Infection Frequency Calculator using the maximum likelihood (ML) method, which showed as IUPM resting CD4^+^ T cells.

### 2.5. PMA and Ionomycin Stimulation Assay

Cryopreserved PBMCs were thawed and recovered in RPMI 1640 supplemented with 10% FBS and 2% penicillin/streptomycin overnight at 37 °C, 5% CO_2_. The PBMCs were stimulated with a Cell Stimulation Cocktail (containing PMA and ionomycin, eBioscience, San Diego, CA, USA). The negative controls were included. Brefeldin A (at a final concentration of 1×; BioLegend, San Diego, CA, USA) and anti-CD107a-BV786 (BD Biosciences, San Jose, CA, USA) were added to all experimental cell wells. Then cells were incubated at 37 °C, 5% CO_2_ for 6 h.

### 2.6. Flow Cytometry

Following stimulation, cells were collected and washed with PBS. Firstly, cells were labeled with the Zombie NIR Fixable Viability Kit (BioLegend) at room temperature for 20 min to distinguish live cells. Then, cells were washed with FACS washing buffer (PBS supplemented with 0.2% BSA and 0.09% sodium azide) and stained with anti-CD3-BV605 (clone SP34-2, BD Biosciences) and anti-CD8-FITC (clone RPAT8, BD Biosciences) at 4 °C for 30 min, then fixed/permeabilized with Fixation and Permeabilization Solution (BD Biosciences) at 4 °C for 20 min. After fixation, cells were washed by Perm/Wash Buffer (BD Biosciences) and stained with anti-IFN-γ-BV711 (clone 4S.B3, BioLegend), anti-MIP-1β-BV421 (clone D21-1351, BD Biosciences), anti-IL-2-PE-Cy7 (clone MQ1-17H12, BioLegend), or anti-TNF-α-BV650 (clone MAb11, BioLegend) at 4 °C for 30 min. Finally, cells were washed by Perm/Wash Buffer and fixed in 1% paraformaldehyde and analyzed on BD FACSAria II within 24 h.

### 2.7. Luminex Detection

The level of plasma cytokines was detected by Luminex assay. Plasma was centrifuged from peripheral blood samples and stored in −80 °C. The following 12 cytokines were measured with a Luminex kit following the manufacturer’s instructions: IFN-γ, IL-2, MCP-1, MIP-1β, IL-1β, IL-6, TNF-α, IL-8, IL-10 (Merck Millipore, Billerica, MA, USA, PRCYTOMAG-40K-09C), IFN-α (Carlsbad, CA, USA, EPX01A-40216-901), IP-10 (Carlsbad, CA, USA, EPX01A-40284-901), and TGF-β (Merck Millipore, Billerica, MA, USA, TGFBMAG-64K-01). Firstly, cryopreserved plasma samples were thawed and mixed completely. A total of 25 μL of plasma samples and 25 μL of assay buffer were added into the corresponding well of 96-well plates and mixed with 25 μL of magnetic beads. The plates wrapped with tinfoil were incubated with agitation for 16–18 h at 4 °C. After incubation, the plates were washed and incubated with 25 μL of detection antibody for 1 h at room temperature (RT). Finally, 25 μL of streptavidin-PE was added to plates and incubated for 30 min at RT. The plates were washed and infiltrated with 150 μL sheath fluid. Plates were loaded on a Luminex^®^ 200 (Bio-Rad, Hercules, CA, USA), and the results were analyzed for median fluorescent intensity using a five-parameter logistic method for calculating analyte concentration.

### 2.8. Statistical Analysis

All data were analyzed using GraphPad Prism 9.0.2 software. Comparisons between the two groups were determined using a two-tailed unpaired Mann-Whitney or *t*-test (Welch).

## 3. Results

### 3.1. Viral Reactivation Generated by Dual NAM and GS–9620 Therapy in Seronegative SHIV_SF162P3_-Infected Rhesus Macaques

As the TLR7 agonist GS–9620 and NAM were potential latency-reversing agents, we determined how those drugs impact the levels of viral reactivation in seronegative SHIV_SF162P3_-infected rhesus macaques. Longitudinal quantification of plasma viral loads demonstrated detectable viral RNA in half of the rhesus macaques treated with GS–9620 and NAM (Figure 1B,C). Two rhesus macaques in the NAM+GS–9620 group separately showed 3.84 Log_10_ RNA copies/mL and 3.99 Log_10_ RNA copies/mL during dual treatment transiently, while all rhesus macaques with GS–9620 monotherapy kept plasma viral loads below the limit of detection (2 Log_10_ RNA copies/mL) (Figure 1B). However, combined drug administration had no evident influence on the level of viral DNA in PBMCs relative to GS–9620 monotherapy (Figure 1D). Additionally, we conducted a TZA assay to quantify inducible replication-competent latent viruses from resting CD4^+^ T cells. As shown in Figure 1E, two monkeys in the NAM+GS–9620 group had 11.06 and 17.07 infectious units per million (IUPM) viable PBMC-derived resting CD4^+^ T cells, with a median value of 7.57 IUPM, which was higher than that of the monkeys in the GS–9620 group (median: 1.37 IUPM). Therefore, dual administration of GS–9620 and NAM was obviously effective in enhancing viral reactivation compared to GS–9620 alone, as demonstrated by both animal-based stratification and the number of detectable events.

### 3.2. CD8^+^ T Cell Responses Induced by Administration of GS–9620 and NAM during Short-Term Therapy

**Figure 2:**
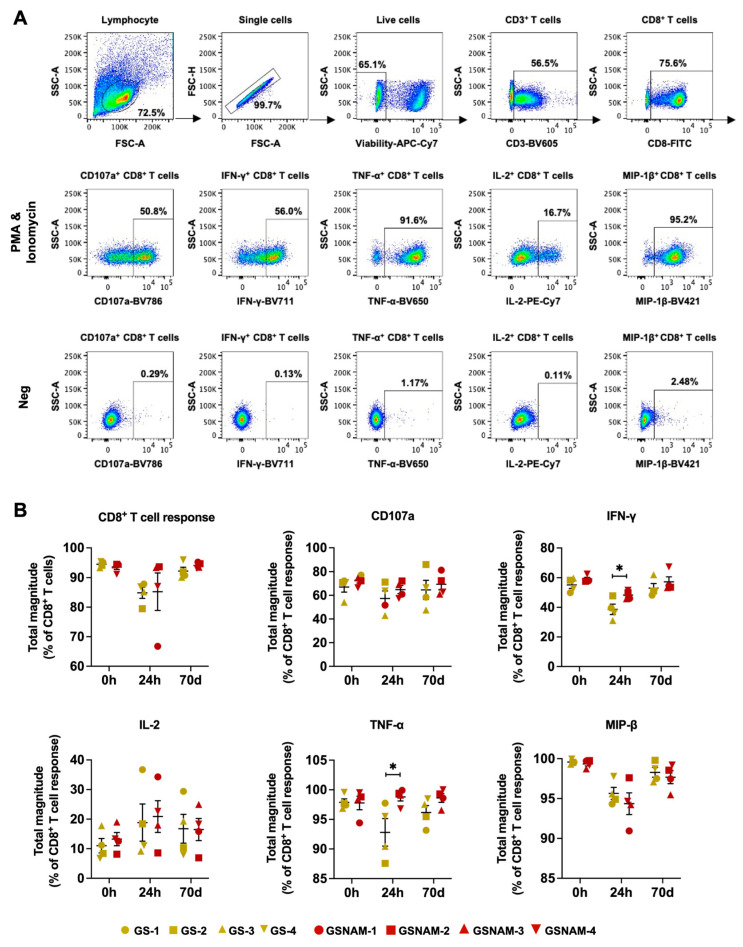
GS–9620 combined with NAM enhanced functionality of CD8^+^ T cells in seronegative SHIV_SF162P3_-infected rhesus macaques in short-term. (**A**) FACS gating scheme for identification of CD8^+^ T cells producing CD107a, IFN-γ, TNF-α, IL-2, and MIP-1β. (**B**) Quantification of cytokine production by CD8^+^ T cells from drug-treated rhesus macaques at 0 h, 24 h, and 70 days after drug administration start. The percentages of CD8^+^ T cells producing CD107a, IFN-γ, TNF-α, IL-2, and MIP-1β were calculated after subtracting the negative group background. The total CD8^+^ T cell response was determined by using the Boolean gate platform based on cytokine production. * p < 0.05.

To determine whether GS–9620 and NAM produce a marked effect on CD8^+^ T cell function, we next measured the CD8^+^ T cell response after PMA and ionomycin stimulation pre-administration (0 d), 24 h (24 h after start), and 70 days post-administration (70 d after start). Flow cytometry analysis revealed that oral administration of GS–9620 and NAM failed to substantially impact the frequency of total CD8^+^ T cells secreting cytokines (CD107a, IFN-γ, TNF-α, IL-2, or MIP-1β). However, as for the single cytokine of CD8^+^ T cells, dual drug therapy increased the expression of IFN-γ (mean: 48.33% vs. 38.66%) and TNF-α (mean: 98.80% vs. 92.82%) in CD8^+^ T cells from PBMCs in SHIV_SF162P3_-infected rhesus macaques in the short-term (24 h, 24 h post-administration) but not in long-term (70 d, 70 days post-administration) relative to GS–9620 monotherapy (Figure 2A,B).

### 3.3. High-Frequency GS–9620 Administration Combined with NAM Therapy Enhances the Reactivation of Replication-Competent Latent Virus in Seronegative SHIV_SF162P3_-Infected Rhesus Macaques

**Figure 3:**
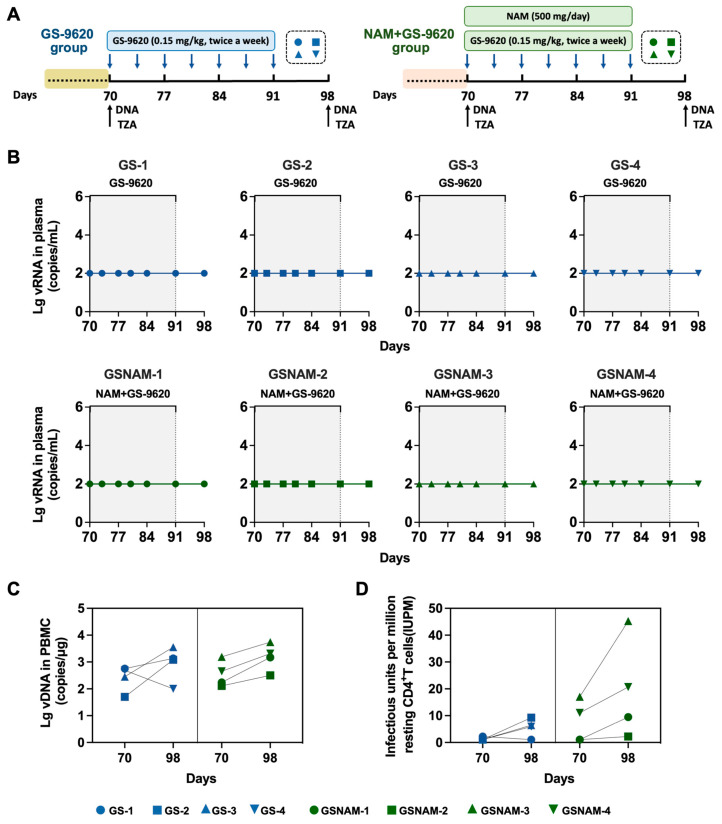
High-frequency GS–9620 administration combined with NAM therapy further reactivated latent virus in seronegative SHIV_SF162P3_-infected rhesus macaques. (**A**) Study design. Rhesus macaques in GS–9620 and NAM+GS–9620 groups continued to be treated with same drug therapy but increasing frequency administration of GS–9620 for 21 days. All rhesus macaques received GS–9620 (0.15 mg/kg) administration twice a week, while macaques in NAM+GS–9620 group were also oral administered NAM (500 mg/day) every day. (**B**) Plasma viral loads of SHIV_SF162P3_-infected monkeys treated with high-frequency drug therapy. The shaded area designates the period for drug treatment. (**C**) Viral DNA in the PBMCs of infected monkeys from two groups at day 70 and 98 after drug administration. (**D**) The frequency of latently infected PBMCs from all infected rhesus macaques at day 70 and 98 after drug therapy.

We have preliminarily observed that dual therapy with GS–9620 and NAM appears to be more effective in in vivo reactivation, as evidenced by viral blips (Figure 1). Next, we further investigated whether increasing the frequency of GS–9620 administration in dual therapy could enhance the possibility of virus reactivation, resulting in a higher frequency of virus rebound in plasma (Figure 3A). Firstly, to determine the influence of the high frequency of GS–9620 in dual treatment on viral reactivation, we measured the levels of viral loads in plasma and viral DNA in PBMCs after adjusting the frequency of GS–9620 administration from biweekly to twice a week. As shown in Figure 3B, high-frequency GS–9620 therapy or its combination with NAM treatment never impacted the level of plasma viral loads, which always were measured as 2 Log_10_ RNA copies/mL. Consistent with the results observed in combined therapy with a low frequency of GS–9620 administration, a high frequency of GS–9620 administration in dual therapy also did not alter viral DNA levels in PBMC samples from SHIV_SF162P3_-infected rhesus macaques compared with GS–9620 monotherapy (Figure 3C). Shockingly, TZA results demonstrated that resting CD4^+^ T cells isolated from PBMCs of the monkeys with a higher frequency of GS–9620 in dual administration had more evident detectable viral outgrowth at day 98 (mean: 16.92 IUPM vs. 5.68 IUPM), which differed from those of the animals treated with a lower frequency of GS–9620 in monotherapy (Figure 3D).

### 3.4. Increased TNF-α Secretion of CD8^+^ T Cells Induced by Treating with High-Frequency GS–9620 Administration Combined with NAM Therapy in Seronegative SHIV_SF162P3_-Infected Rhesus Macaques

**Figure 4:**
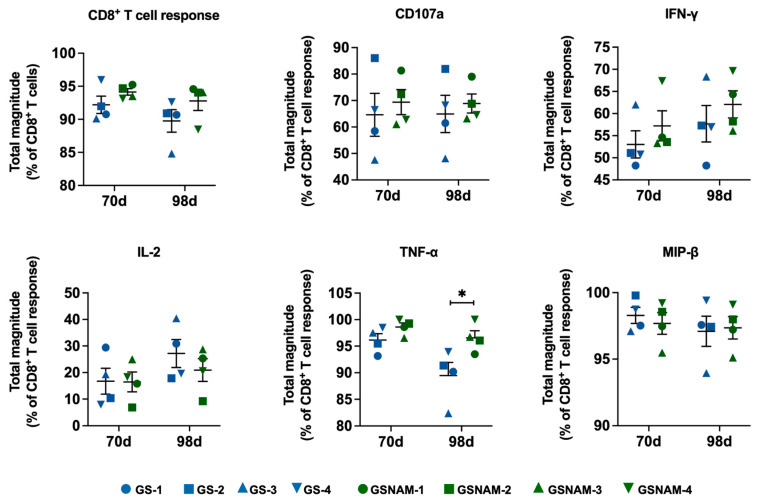
High-frequency administration of GS–9620 combined with NAM therapy promoted TNF-α secretion of CD8^+^ T cells in seronegative SHIV_SF162P3_-infected rhesus macaques. The percentages of CD8^+^ T cells producing CD107a, IFN-γ, TNF-α, IL-2, and MIP-1β were calculated after background subtraction at day 70 and 98 post drug treatment. The total CD8^+^ T cell response was determined by using the Boolean gate platform dependent on cytokine production. * p < 0.05.

**Figure 5:**
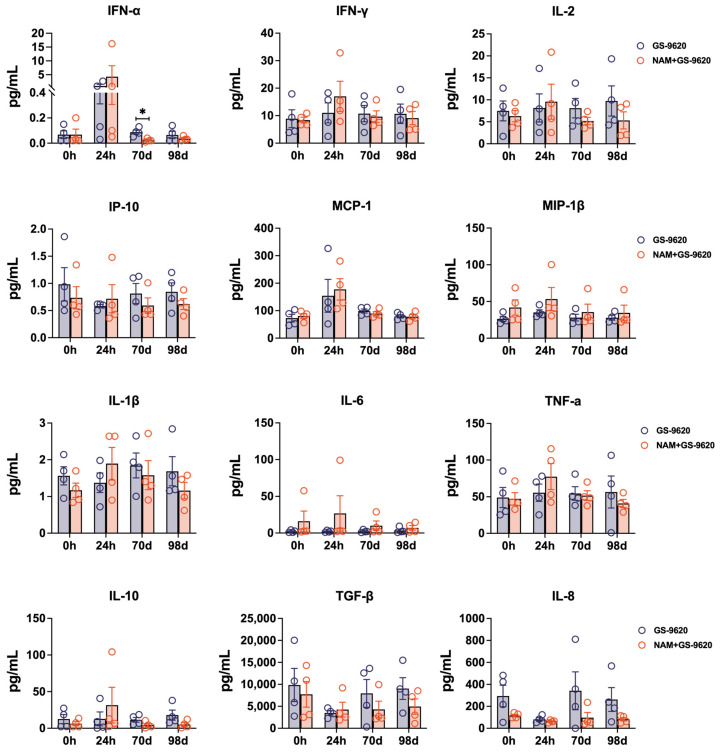
Dynamics of cytokines in plasma of seronegative SHIV_SF162P3_-infected rhesus macaques treated with GS–9620 or combined with NAM. The level of cytokines in plasma was measured by Luminex detection at two drug treatment stages with different frequency of administration of GS–9620. * *p* < 0.05.

To assess if a high frequency of GS–9620 in dual therapy could favor the formation of superior CD8^+^ T cell functionality, we assayed for cytokine production of CD8^+^ T cells stimulated with PMA and ionomycin before and after therapy with high-frequency GS–9620 administration. The proportion of total functional CD8^+^ T cells (CD8^+^ T cells expressing CD107a, IFN-γ, TNF-α, IL-2, or MIP-1β) was remarkably stable in GS–9620 monotherapy and dual therapy after increasing the frequency of GS–9620 administration (GS–9620 monotherapy, mean: 89.76%; dual therapy, mean: 92.80%, day 98, Figure 4). In comparison, high-frequency GS–9620 in dual therapy resulted in the upregulation of TNF-α expression in CD8^+^ T cells at 98 days, which was most pronounced relative to monotherapy (96.57% vs. 89.47%, *p* < 0.05, day 98, Figure 4). However, the approximate level of various cytokines (IFN-α, IFN-γ, IL-2, IP-10, MCP-1, MIP-1β, IL-1β, IL-6, TNF-α, IL-10, TGF-β, and IL-8) in plasma was not affected by these drug treatments (Figure 5). We only observed that SHIV_SF162P3_-infected rhesus macaques treated with GS–9620 biweekly for 70 days had higher levels of IFN-α in plasma relative to dual treatment (Figure 5).

## 4. Discussion

The major obstacle to achieving a functional cure for HIV is the establishment of long-lasting and replication-competent latent reservoirs [23]. The “shock and kill” strategy for an HIV cure primarily focuses on controlling the infection by inducing viral latency activation using potent LRAs, subsequently allowing antiviral therapy or the host immune system to recognize and eliminate the residual infected cells [1,7]. GS–9620, a selective oral TLR7 agonist developed by Gilead, is currently among the promising LRAs under investigation [10,13,24,25]. Both rhesus macaque experiments and human clinical trials have demonstrated GS–9620′s ability to reactivate latent viral reservoirs and induce transient viremia under ART [11,12]. Another promising LRA, NAM, when combined with ART, has demonstrated potential effectiveness in curing HIV, as evidenced by the treatment of the “Sao Paulo patient”, yielding an encouraging response.

In this study, we demonstrated the enhanced efficacy of combined GS–9620 and NAM therapy in reversing viral reservoirs in the nonhuman primate model of HIV spontaneous control, in the absence of antiviral therapy. Furthermore, we observed a higher IUPM of isolated resting CD4^+^ T cells in the peripheral blood of animals in the NAM+GS–9620 group compared to the GS–9620 group, suggesting that the combination of NAM and GS–9620 may exert synergistic effects in activating latent infected cells. Interestingly, previous studies have reported that GS–9620 induces the secretion of TNF-α and IFN-α by plasmacytoid dendritic cells (pDCs) or TNF-α by monocytes, which is necessary for T cell activation and subsequent latent viral reactivation in CD4^+^ T cells [10,26]. As for NAM, it acts as a deacetylase inhibitor that blocks SIRT1 (a class III HDAC), thereby inhibiting histone deacetylation and subsequent activation of NF-κB, resulting in promotor activation and latency disruption [27,28,29]. Consequently, we speculate that the synergistic effects of GS–9620 and NAM on virus activation may be attributed to both inflammatory factor (TNF-α and IFN-α) secretion and SIRT1 inhibition. The potential synergistic role of these two agents in latency reversal in vivo remains to be determined.

TLR7 agonists can stimulate plasmacytoid dendritic cells (pDCs) to produce type I interferons (IFNs, such as IFN-α and IFN-β), which activate NK cells. Subsequently, activated NK cells can eliminate virus-infected cells by augmenting CD8^+^ T function [30,31], thereby mediating both innate and adaptive immunity [32,33]. Our study found that administering a combination therapy of GS–9620 and NAM led to a significant increase in IFN-γ and TNF-α expression on CD8^+^ T cells in SHIV-infected monkeys for a short duration. Additionally, increasing the frequency of GS–9620 administration in the combined therapy further promoted TNF-α secretion by CD8^+^ T cells. Thus, we speculate that the immunity of CD8^+^ T cells may be enhanced by cytokines secreted from intrinsic immune cells stimulated by GS–9620 and NAM [10]. However, it is worth noting that this non-virus specific CD8^+^ T cell function only partially reflects the immune killing ability of CD8^+^ T cells. It would be more appropriate to determine polyfunctional SHIV-specific CD8^+^ T cells with virus-inhibiting or virus-lytic potential, which could indeed constitute the highly desired “kill” component in the “shock and kill” strategy. Additionally, when combined with the results regarding total DNA during treatment, the dual therapy exhibited a promising influence on virus reversal. However, the total proviral DNA level in PBMCs of SHIV-infected monkeys did not change significantly. This observation could be attributed to our measurement for proviral DNA, which could not separately quantify intact and defective proviruses. Therefore, the use of the intact proviral DNA assay (IPDA), a more accurate method of measuring the virus reservoir that distinguishes genomically intact proviruses, needs to be considered. Alternatively, another possibility is that the SHIV-specific CD8^+^ T cell immunity was not sufficiently enhanced to eliminate the reactivated virus and reduce virus reservoirs.

Several limitations of this study should be acknowledged. First, the sample size of rhesus macaques could be increased to obtain more convincing findings. Second, further research is necessary to determine SHIV-specific CD8^+^ T cell ployfunctions and assess killing efficacy during GS–9620 and NAM therapy. Third, the size and reactivation of tissues’ viral reservoirs, besides PBMCs, also need to be determined.

## 5. Conclusions

Taken together, our results demonstrate that the combination of GS–9620 and NAM therapy exhibits viral blips in plasma and the enhancement of the reactivation of replication-competent latent viruses in aviremic SHIV_SF162P3_-infected rhesus macaques, and it also modestly stimulates CD8^+^ T cell effector functions. The safety and antiviral activity observed with GS–9620 and NAM co-administration could help offer novel orientation for ongoing HIV animal model experiments and preclinical studies.

## Figures and Tables

**Table 1:** Basic information for rhesus macaque challenged by SHIV_SF162P3_.

Group		GS–9620				NAM+GS–9620			
Animal No. in this study		GS-1	GS-2	GS-3	GS-4	GSNAM-1	GSNAM-2	GSNAM-3	GSNAM-4
Gender		Male	Male	Male	Male	Male	Male	Male	Male
Weight (kg)		11.90	8.10	9.20	7.80	10.80	8.10	9.50	9.50
Alleles associated with the control of SIV/SHIV replication	MHC-Ⅰ Mamu-A*01	NEG	NEG	NEG	NEG	NEG	NEG	NEG	NEG
	MHC-Ⅰ Mamu-A*02	NEG	NEG	NEG	NEG	NEG	NEG	NEG	NEG
	MHC-Ⅰ Mamu-B*08	NEG	NEG	NEG	NEG	NEG	NEG	NEG	NEG
	MHC-Ⅰ Mamu-B*12	NEG	NEG	NEG	NEG	NEG	NEG	NEG	NEG
Pathogen	Simian immunodeficiency virus	NEG	NEG	NEG	NEG	NEG	NEG	NEG	NEG
	Simian type D retrovirus	NEG	NEG	NEG	NEG	NEG	NEG	NEG	NEG
	Simian T-lymphotropic virus	NEG	NEG	NEG	NEG	NEG	NEG	NEG	NEG
	Herpes B virus	NEG	NEG	NEG	NEG	NEG	NEG	NEG	NEG
SHIV_SF162P3_ Challenge	Route	*I.R.*	*I.R.*	*I.R.*	*I.R.*	*I.R.*	*I.R.*	*I.R.*	*I.R.*
	Inoculum (TCID_50_)	500	500	500	500	500	500	500	500
	Challenge strategy	Mucosal exposure with SHIV_SF162P3_ twice-weekly for three weeks				Mucosal exposure with SHIV_SF162P3_ twice-weekly for three weeks			

MHC-Ⅰ, major histocompatibility complex class I; *I.R.*, intrarectal route; NEG, negative.

## Data Availability

The datasets generated and/or analyzed during this study are available from the corresponding author upon reasonable request.

## References

[1] Kim Y., Anderson J.L., Lewin S.R. (2018). Getting the “Kill” into “Shock and Kill”: Strategies to Eliminate Latent HIV. Cell Host Microbe.

[2] Menendez-Arias L., Delgado R. (2022). Update and latest advances in antiretroviral therapy. Trends Pharmacol. Sci..

[3] Sadowski I., Hashemi F.B. (2019). Strategies to eradicate HIV from infected patients: Elimination of latent provirus reservoirs. Cell Mol. Life Sci..

[4] Churchill M.J., Deeks S.G., Margolis D.M., Siliciano R.F., Swanstrom R. (2016). HIV reservoirs: What, where and how to target them. Nat. Rev. Microbiol..

[5] Barton K., Winckelmann A., Palmer S. (2016). HIV-1 Reservoirs During Suppressive Therapy. Trends Microbiol..

[6] Abner E., Jordan A. (2019). HIV “shock and kill” therapy: In need of revision. Antiviral Res..

[7] Kula-Pacurar A., Rodari A., Darcis G., Van Lint C. (2021). Shocking HIV-1 with immunomodulatory latency reversing agents. Semin. Immunol..

[8] Martinsen J.T., Gunst J.D., Hojen J.F., Tolstrup M., Sogaard O.S. (2020). The Use of Toll-Like Receptor Agonists in HIV-1 Cure Strategies. Front. Immunol..

[9] Macedo A.B., Novis C.L., Bosque A. (2019). Targeting Cellular and Tissue HIV Reservoirs With Toll-Like Receptor Agonists. Front. Immunol..

[10] Tsai A., Irrinki A., Kaur J., Cihlar T., Kukolj G., Sloan D.D., Murry J.P. (2017). Toll-Like Receptor 7 Agonist GS-9620 Induces HIV Expression and HIV-Specific Immunity in Cells from HIV-Infected Individuals on Suppressive Antiretroviral Therapy. J. Virol..

[11] SenGupta D., Brinson C., DeJesus E., Mills A., Shalit P., Guo S., Cai Y., Wallin J.J., Zhang L., Humeniuk R. (2021). The TLR7 agonist vesatolimod induced a modest delay in viral rebound in HIV controllers after cessation of antiretroviral therapy. Sci. Transl. Med..

[12] Lim S.Y., Osuna C.E., Hraber P.T., Hesselgesser J., Gerold J.M., Barnes T.L., Sanisetty S., Seaman M.S., Lewis M.G., Geleziunas R. (2018). TLR7 agonists induce transient viremia and reduce the viral reservoir in SIV-infected rhesus macaques on antiretroviral therapy. Sci. Transl. Med..

[13] Bam R.A., Hansen D., Irrinki A., Mulato A., Jones G.S., Hesselgesser J., Frey C.R., Cihlar T., Yant S.R. (2017). TLR7 Agonist GS-9620 Is a Potent Inhibitor of Acute HIV-1 Infection in Human Peripheral Blood Mononuclear Cells. Antimicrob. Agents Chemother..

[14] Walker-Sperling V.E.K., Mercado N.B., Chandrashekar A., Borducchi E.N., Liu J., Nkolola J.P., Lewis M., Murry J.P., Yang Y., Geleziunas R. (2022). Therapeutic efficacy of combined active and passive immunization in ART-suppressed, SHIV-infected rhesus macaques. Nat. Commun..

[15] Borducchi E.N., Liu J., Nkolola J.P., Cadena A.M., Yu W.H., Fischinger S., Broge T., Abbink P., Mercado N.B., Chandrashekar A. (2018). Antibody and TLR7 agonist delay viral rebound in SHIV-infected monkeys. Nature.

[16] Ventura J.D., Nkolola J.P., Chandrashekar A., Borducchi E.N., Liu J., Mercado N.B., Hope D.L., Giffin V.M., McMahan K., Geleziunas R. (2022). Therapeutic efficacy of an Ad26/MVA vaccine with SIV gp140 protein and vesatolimod in ART-suppressed rhesus macaques. NPJ Vaccines.

[17] Moldt B., Chandrashekar A., Borducchi E.N., Nkolola J.P., Stephenson H., Nagel M., Hung M., Goldsmith J., Pace C.S., Carr B. (2022). HIV envelope antibodies and TLR7 agonist partially prevent viral rebound in chronically SHIV-infected monkeys. PLoS Pathog..

[18] Samer S., Arif M.S., Giron L.B., Zukurov J.P.L., Hunter J., Santillo B.T., Namiyama G., Galinskas J., Komninakis S.V., Oshiro T.M. (2020). Nicotinamide activates latent HIV-1 ex vivo in ART suppressed individuals, revealing higher potency than the association of two methyltransferase inhibitors, chaetocin and BIX01294. Braz. J. Infect. Dis..

[19] Bricker K.M., Chahroudi A., Mavigner M. (2021). New Latency Reversing Agents for HIV-1 Cure: Insights from Nonhuman Primate Models. Viruses.

[20] Delagreverie H.M., Delaugerre C., Lewin S.R., Deeks S.G., Li J.Z. (2016). Ongoing Clinical Trials of Human Immunodeficiency Virus Latency-Reversing and Immunomodulatory Agents. Open. Forum Infect. Dis..

[21] Chong H., Xue J., Zhu Y., Cong Z., Chen T., Wei Q., Qin C., He Y. (2019). Monotherapy with a low-dose lipopeptide HIV fusion inhibitor maintains long-term viral suppression in rhesus macaques. PLoS Pathog..

[22] Xue J., Chong H., Zhu Y., Zhang J., Tong L., Lu J., Chen T., Cong Z., Wei Q., He Y. (2022). Efficient treatment and pre-exposure prophylaxis in rhesus macaques by an HIV fusion-inhibitory lipopeptide. Cell.

[23] Sengupta S., Siliciano R.F. (2018). Targeting the Latent Reservoir for HIV-1. Immunity.

[24] Fosdick A., Zheng J., Pflanz S., Frey C.R., Hesselgesser J., Halcomb R.L., Wolfgang G., Tumas D.B. (2014). Pharmacokinetic and pharmacodynamic properties of GS-9620, a novel Toll-like receptor 7 agonist, demonstrate interferon-stimulated gene induction without detectable serum interferon at low oral doses. J. Pharmacol. Exp. Ther..

[25] Roethle P.A., McFadden R.M., Yang H., Hrvatin P., Hui H., Graupe M., Gallagher B., Chao J., Hesselgesser J., Duatschek P. (2013). Identification and optimization of pteridinone Toll-like receptor 7 (TLR7) agonists for the oral treatment of viral hepatitis. J. Med. Chem..

[26] Macedo A.B., Novis C.L., De Assis C.M., Sorensen E.S., Moszczynski P., Huang S.H., Ren Y., Spivak A.M., Jones R.B., Planelles V. (2018). Dual TLR2 and TLR7 agonists as HIV latency-reversing agents. JCI Insight.

[27] Khan J.A., Forouhar F., Tao X., Tong L. (2007). Nicotinamide adenine dinucleotide metabolism as an attractive target for drug discovery. Expert. Opin. Ther. Targets.

[28] Kwon H.S., Brent M.M., Getachew R., Jayakumar P., Chen L.F., Schnolzer M., McBurney M.W., Marmorstein R., Greene W.C., Ott M. (2008). Human immunodeficiency virus type 1 Tat protein inhibits the SIRT1 deacetylase and induces T cell hyperactivation. Cell Host Microbe.

[29] Pagans S., Pedal A., North B.J., Kaehlcke K., Marshall B.L., Dorr A., Hetzer-Egger C., Henklein P., Frye R., McBurney M.W. (2005). SIRT1 regulates HIV transcription via Tat deacetylation. PLoS Biol..

[30] Takeuchi O., Akira S. (2010). Pattern recognition receptors and inflammation. Cell.

[31] Ram R.R., Duatschek P., Margot N., Abram M., Geleziunas R., Hesselgesser J., Callebaut C. (2020). Activation of HIV-specific CD8(+) T-cells from HIV+ donors by vesatolimod. Antivir. Ther..

[32] Iwasaki A., Medzhitov R. (2004). Toll-like receptor control of the adaptive immune responses. Nat. Immunol..

[33] Luchner M., Reinke S., Milicic A. (2021). TLR Agonists as Vaccine Adjuvants Targeting Cancer and Infectious Diseases. Pharmaceutics.

